# The Effect of Temperature and Strain Rate on the Interfacial Behavior of Glass Fiber Reinforced Polypropylene Composites: A Molecular Dynamics Study

**DOI:** 10.3390/polym11111766

**Published:** 2019-10-27

**Authors:** Muhan Zhang, Bingyan Jiang, Chao Chen, Dietmar Drummer, Zhanyu Zhai

**Affiliations:** 1 State Key Laboratory of High Performance and Complex Manufacturing, Light Alloy Research Institute, Central South University, Lushan South Road 932, Changsha 410083, China; 183811004@csu.edu.cn (M.Z.); zzyu1021@hotmail.com (B.J.); profchenchao@163.com (C.C.); 2 Friedrich-Alexander-University Erlangen-Nürnberg, Institute of Polymer Technology, Am Weichselgarten 9, D-91058 Erlangen, Germany; drummer@lkt.uni-erlangen.de

**Keywords:** interface, polymer-matrix composites (PMCs), adhesion, molecular dynamics simulation

## Abstract

To make better use of fiber reinforced polymer composites in automotive applications, a clearer knowledge of its interfacial properties under dynamic and thermal loadings is necessary. In the present study, the interfacial behavior of glass fiber reinforced polypropylene (PP) composites under different loading temperatures and strain rates were investigated via molecular dynamics simulation. The simulation results reveal that PP molecules move easily to fit tensile deformation at higher temperatures, resulting in a lower interfacial strength of glass fiber–PP interface. The interfacial strength is enhanced with increasing strain rate because the atoms do not have enough time to relax at higher strain rates. In addition, the non-bonded interaction energy plays a crucial role during the tensile deformation of composites. The damage evolution of glass fiber–PP interface follows Weibull’s distribution. At elevated temperatures, tensile loading is more likely to cause cohesive failure because the mechanical property of PP is lower than that of the glass fiber–PP interface. However, at higher strain rates, the primary failure mode is interfacial failure because the strain rate dependency of PP is more pronounced than that of the glass fiber–PP interface. The relationship between the failure modes and loading conditions obtained by molecular dynamics simulation is consistent with the author’s previous experimental studies.

## 1. Introduction

With the increasing demand for eco-friendly transportation systems, lightweight continuous fiber reinforced thermoplastic composites (CFRTCs) have attracted great interest in the automotive industry due to their specific properties, such as excellent corrosion resistance, superior mechanical properties, and high strength-to-weight ratio [1,2,3]. Composite structures for automotive applications are often subjected to many different and complex loading conditions, including highly dynamic and thermomechanical loadings [4,5]. The mechanical properties of CFRTCs are related to the properties of reinforcing fiber, matrix, and fiber–matrix interfaces. Rafiee et al. [6,7] reported that the simultaneous enhancement of fiber and matrix could benefit the materials of laminated composites. Meanwhile, the morphological features of the matrix may enhance the stress transfer capability across the fiber–matrix interface [8]. Generally, most of the commonly applied thermoplastic matrix systems are sensitive to loading rate and temperature, thus also affecting the resulting composites properties and its interface. According to the interface transfer theory [9], the fiber–matrix interface is responsible for the mechanical behavior of composites by affecting the transfer of load from the matrix to the fibers. Furthermore, it is found that the damage of composites structures most often initiates and propagates from the changes at the fiber–matrix interface [10,11]. Obviously, the fiber–matrix interfacial property severely restricts the design and application of CFRTCs. Therefore, to make a better use of CFRTCs in automotive area, changes in the fiber–matrix interfacial property under highly dynamic and thermal loadings must be understood.

**Figure 1:**
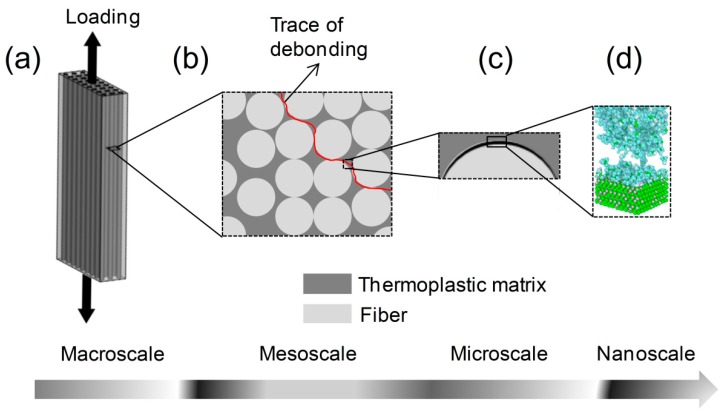
Structural hierarchy of damaged glass fiber reinforced thermoplastic composites from macro- to nanoscale: (**a**) macroscale composite sample; (**b**) mesoscale cross-section; (**c**) microscale fiber–matrix interface with size down to several nanometers; and (**d**) nanoscale interface with matrix molecule and amorphous SiO_2_.

So far, considerable attentions [4,12,13,14,15] have been paid on the overall mechanical properties of bulk composites with respect to the different loading conditions, like loading temperature and strain rate. The loading mode is shown in Figure 1a. They demonstrated that the matrix-dominated properties and failure mechanisms of bulk composites are significantly temperature and strain rate dependent. Meanwhile, micromechanical test methods, such as the micro-bond method, the single fiber pull-out test and the single fiber fragmentation test, were also performed on micro-CFRTCs (as shown in Figure 1c) to obtain the interfacial shear strength of composites under various loading conditions. The results achieved from these studies [16,17,18,19] suggest that the fiber–matrix interface is particularly vulnerable to loading temperatures and rates. The previous investigations have provided useful information on the interfacial properties of CFRTCs about temperature dependency and strain rate dependency through using scanning electron microscopy (SEM), atomic force microscopy (AFM), micro- and macromechanical test methods, but some limitations and challenges have still yet to be solved. It is well known that the size of fiber–matrix interphase region is down to several nanometers, as shown in Figure 1d. Thus, it is difficult to identify the properties changes at the fiber–matrix interface at the molecular scale due to the current limitations in experimental techniques.

In recent years, molecular dynamics (MD) simulation has been increasingly employed to investigate the fiber–matrix interface property of composites from a viewpoint of atomistic level. Niuchi et al. [20] investigated the tensile stress and fracture energy between phenolic resin and carbon fiber with different surface properties through the MD method. Koyanagi and co-workers [21] evaluated the mechanical properties of three types of interface, i.e., carbon/vinyl ester resin, carbon fiber/epoxy resin, and carbon fiber/polyimide resin via a microbonding test and normal stretching MD simulations. They claimed that the experimentally-obtained interfacial strengths of three systems followed the same orders as the numerically-obtained interfacial energies. However, the numerically-obtained interfacial strength tends to overestimate the experimental values. Tam et al. [22,23] investigated the interfacial properties and debonding process of a carbon fiber–epoxy interface in hygrothermal conditioning. Their findings contribute to the nanoscale insight into interfacial deterioration mechanism of CFRTCs in hygrothermal environment. The above studies have provided meaningful information about the interactions between fiber and matrix at the molecular level. Nevertheless, to the authors’ knowledge, the microscopic details of interfacial structure, stress, and debonding process of the fiber–matrix interface under different loading temperatures and strain rates have not been understood.

The objective of this paper is to investigate the interfacial mechanical behavior of a glass fiber–polypropylene interface (glass fiber–PP interface) of composites exposed to different loading temperatures and strain rates by using MD simulation. The molecular interface model consisting of glass fiber, a silane sizing layer, and polypropylene matrix is firstly built. Uniaxial tensile deformation simulations are performed on the interface models. The stress–strain response, density, interfacial energy, and fracture energy of the glass fiber–PP interface are estimated. Fracture mechanisms associated with loading parameters are also investigated during tension process.

## 2. Simulation Model and Methodology

### 2.1. Molecular Model and Force Field

#### 2.1.1. Model Constructing

Polypropylene (PP) is one of the most promising matrix materials for CFRTCs used in automotive area because it possesses excellent specific properties, like high price-performance ratio, low processing temperature, and low water absorption [24]. Thus, in this study a PP (Moplen EP500V) supplied by LyondellBasell company (Houston, TX, USA), was chosen as a matrix for CFRTCs. The melting temperature of PP resin is 168 °C. The reinforcement for CFRTCs is the E-glass fabric provided by P-D Glasseiden GmbH (Oschatz, Germany). The silane coupling agent of γ-Aminopropyltriethoxysilane (*γ*-APS) was selected as glass fiber sizing.

**Figure 2:**
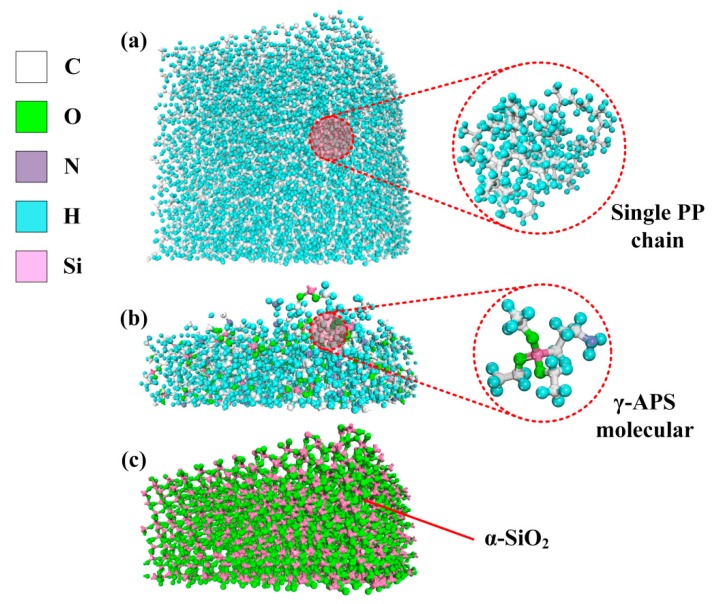
Atomistic models of (**a**) PP matrix, (**b**) silane sizing, and (**c**) glass fiber.

**Figure 3:**
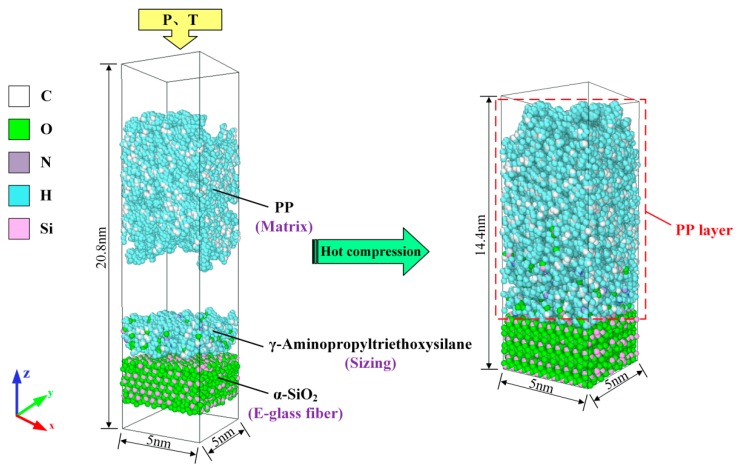
Molecular dynamics (MD) visualization of glass fiber–PP interface model.

Before constructing the fiber–PP interface model, atomistic models of PP matrix, silane sizing, and glass fiber were separately constructed, as shown in Figure 2. PP matrix was built as a rectangular cuboid with dimensions 5 × 5 nm^2^ in length and width. The other important settings about PP matrix, including the number of chains, degree of polymerization, total amount of atoms, initial density, and box size, are listed in Table 1. The box size of sizing layer was set as 5 × 5 × 1.56 nm^3^ with 100 *γ*-APS molecules distributed randomly. Taking the same approach with references [25,26], the amorphous SiO_2_ (a-SiO_2_) structure was employed to represent glass fiber at the atomic level. The dimensions of glass fiber were 5 × 5 × 2.49 nm^3^. In order to obtain an irregular arrangement of SiO_2_ atom model to match the actual situation closely, a melt-anneal procedure was used to simulate a 5400-atom-crystalline amorphous SiO_2_ (*α*-SiO_2_) structure at the temperature of 2000 K, following by some suitable operations to optimize the spatial structure of the molecule. Finally, boxes of glass fiber, silane sizing, PP matrix, and a vacuum layer with a thickness of 2 nm were sequentially placed in the order from bottom to top to form a complete simulation system. The size of the initial simulation box is 5 × 5 × 20.8 nm^3^, as shown in Figure 3. The periodic boundary conditions (PBC) were applied to the *x*- and *y*-axis to replicate the large aspect ratio of polymer particles, while the free boundary conditions were applied to the *z*-axis so that the whole system can be effectively compressed during the following process.

Glass fiber reinforced polypropylene composites (glass fiber/PP composites) was produced by hot compression technique. The processing parameters in this simulation work were set in accordance with our previous experimental study [27]. To be specific, hot compression was applied on the initial simulation system under the pressure of 1 MPa and the temperature of 210 °C. The fiber–PP interface was formed after the whole system was cooled to room temperature, as shown in Figure 3. The dimensions of the complete, relaxed fiber–matrix interface model were 5 × 5 × 14.4 nm^3^.

#### 2.1.2. Force Field

The intermolecular and non-bonded interactions between atoms in the glass fiber–PP interface were described by the consistent valence force field (CVFF), which has been gradually applied to a variety of polypeptides, proteins, and a large number of organic molecular systems. In recent years, CVFF has been broadly used to study the interfacial properties of polymer-substrate interfaces [28,29]. In this work, bonding stretching potential, angular bending potential, and torsion potential were chosen. Standard Lennard–Jones 12-6 potential and Coulomb pairwise interaction were employed to describe the non-bond interactions between glass fiber and PP. The energy function of CVFF potential is shown as Equation (1).

(1) $$E=E_{\mathrm{bond}}+E_{\mathrm{angle}}+E_{\mathrm{torsion}}+E_{\mathrm{improper}}+E_{\mathrm{vdw}}+E_{\mathrm{coul}}\;\;\;\;\;\;=\underset{\mathrm{bond}}{\sum }K_{b}{\left(b-b_{0}\right)}^{2}+\underset{\mathrm{angle}}{\sum }K_{a}{\left(\theta -\theta_{0}\right)}^{2}+\underset{torsion}{\sum }K_{t}\left[1+s\cos\left(n\emptyset_{t}\right)\right]\;\;\;\;\;\;\;+\underset{\mathrm{improper}}{\sum }K_{o}\left[1+s\cos\left(n\emptyset_{i}\right)\right]+\underset{ij}{\sum }4\varepsilon_{ij}\left[{\left(\frac{\sigma_{ij}}{r}\right)}^{12}-{\left(\frac{\sigma_{ij}}{r}\right)}^{6}\right]+\underset{ij}{\sum }\frac{q_{i}q_{j}}{\varepsilon r_{ij}}$$

where $E_{\mathrm{bond}}$, $E_{\mathrm{angle}}$, $E_{\mathrm{torsion}}$, $E_{\mathrm{improper}}$ are the functional term of bond stretching, angle bending, dihedral angle torsion, respectively. $E_{improper}$ is the improper of the bonded interaction. $E_{\mathrm{vdw}}$ and $E_{coul}$ are the van der Waals and Coulombic, respectively. In Equation (1), the $K_{b}$, $K_{a}$, $K_{t}$ and ε are the constants of the bond stretching potential, angular bending potential, torsion potential, and non-bonded interaction, respectively. Parameters are derived from the lattice constant and adhesive energy of SiO_2_ atom. The cut-off distance of both potentials in non-bonded interaction is 1.25 nm.

### 2.2. Dynamics Simulations

In this work, all the MD simulations were performed using the open source code LAMMPS (large-scale atomic/molecular massively parallel simulation) [30].

#### 2.2.1. Equilibration Stage

To get an equilibrium structure of a fiber–PP interface, a series of simulations were taken. At the beginning, similar procedure was repeated on fiber and PP molecules using three algorithms, steepest descent, conjugate gradient and Newton methods with 10,000 iterations, which can minimize the total potential energy of glass fiber/PP composites system. Specially, the PP box was annealed from 300 to 500 K and then back to 300 K with a temperature gradient of 40 K/ps, leading to a lower overall stress. It is necessary to rebalance the total energy of composites system before starting uniaxial tensile simulations, which could improve the accuracy of calculation results. Hence, the glass fiber/PP composites system was firstly relaxed in a microcanonical NVE ensemble with the Langevin method for 20 ps (∆*t* = 1 fs). Subsequently, the system was relaxed in an isothermal-isobaric NPT ensemble at a target temperature and 1 atm for 40 ps (∆*t* = 1 fs). The purpose of this step is to achieve a stress-free state.

#### 2.2.2. Uniaxial Tensile Deformation

Followed by equilibration, a series of uniaxial tensile simulations were conducted to determine the tensile response of fiber–PP interface under different loading temperatures and strain rates. To be specific, the tensile behavior at the loading temperatures of 250 K, 298 K, 363 K, 403 K, and the strain rate of 2 × 10^11^ s^−1^ were carried on to investigate the effect of loading temperature. Four strain rates, 1 × 10^11^ s^−1^, 2 × 10^11^ s^−1^, 3 × 10^11^ s^−1^, and 4 × 10^11^ s^−1^ were chosen to study the effect of strain rates at the loading temperature of 298 K. During tensile process, an isothermal-isobaric NPT ensemble was applied with the temperature kept at loading temperature correspondingly for 10 ps (∆*t* = 0.1 fs). In addition, the stress–strain curves obtained from the above simulations were smoothed using the Savitzky–Golay method to eliminate the fluctuation.

### 2.3. The mobility of Polymer Molecular Chains

The mean square displacement (MSD) and radius of gyration ($R_{g}$) are adopted to characterize the mobility of polymer molecules and chains, which are calculated by Equations (2) and (3) respectively,

(2) $$\mathrm{MSD}=6t\langle {\left|r_{i}\left(t\right)-r_{i}\left(0\right)\right|}^{2}\rangle$$

In the equation, $r_{i}\left(t\right)$ and $r_{i}\left(0\right)$ represent the position vectors of the first atom at time t and zero, respectively.

(3) $$R_{g}=\sqrt{\frac{1}{M}\underset{i}{\sum }m_{i}{\left(r_{i}-r_{cm}\right)}^{2}}$$

where *M* is the total mass of the polymer chains in the simulation system and $r_{cm}$ is the center-of-mass position of the chains. It is assumed that a plurality of chain units is contained in a polymer chain. The mass of each chain unit is $m_{i}$ and $r_{i}$ is the distance of an atom to the center-of-mass position of a single chain.

### 2.4. Interfacial Bonding Energy

The chemical bonding theory was adopted to study the fiber–PP interface of composites in order to explore how the interactions between molecules affect the formation of interface. The value of the interface energy was used to assess the interfacial bonding strength. High energy values indicate strong interfacial adsorption and stable structure. The interfacial energy was calculated by the following Equation (4):

(4) $$E_{\mathrm{interfacial}}=-E_{\mathrm{inter}}=-\left(E_{\mathrm{GFRP}}-\left(E_{\mathrm{polymer}}+E_{\mathrm{fiber}}\right)\right)$$

where $E_{\mathrm{interfacial}}$ is the interfacial energy of virgin fiber–matrix interface, while $E_{\mathrm{GFRP}}$, $E_{\mathrm{polymer}}$ and $E_{\mathrm{fiber}}$ are the energy of the whole system, polymer, and E-glass fiber box, respectively.

### 2.5. Interfacial Fracture Energy

Interfacial fracture energy is defined as the difference in energy of fiber–PP interface between the adhesive states and detachment state [20], as follows:

(5) $$E_{\mathrm{fracture}}=\frac{E_{\mathrm{total}}^{\mathrm{adhesive}}-E_{\mathrm{total}}^{\mathrm{detachment}}}{A_{\mathrm{contact}}}$$

where $E_{\mathrm{total}}^{\mathrm{adhesive}}$ and $E_{\mathrm{total}}^{\mathrm{detachment}}$ denote the total energy of composites system at adhesive state and detachment state, respectively. The total energy of adhesive state can be obtained through Equation (4). $A_{\mathrm{contact}}$ indicates the effective contact area between polymer and fiber layer. The difference in energy between two states was divided by the effective contact area, which is to convert the interfacial fracture from a unit of kcal/mol to J/m^2^.

### 2.6. Damage Evolution at the Microscopic Scale

To evaluate the temperature and strain rate dependence of the damage growth in glass fiber/PP composites, the damage analysis was conducted at the microscopic scale. In terms of ply level, it is assumed that the damage events are composed of matrix crack, fiber–matrix interface debonding and fiber breakage [31]. Generally, the fiber damage leads to ultimate failure of composites. The damage events of matrix crack and fiber–matrix interface debonding are related to the ply damage evolution. The microscopic damage was quantified by the density reduction of the glass fiber/PP composites system during tensile loading. The damage scalar variable D(t) is proposed as

(6) $$D\left(t\right)=1-\frac{\rho \left(t\right)}{\rho_{0}}$$

where t is the loading time; $\rho_{0}$ and ρ(t) are the density of virgin and damaged glass fiber/PP composites, respectively.

## 3. Results and Discussion

The results obtained from MD simulations are given here, including the interfacial mechanical response of the fiber–PP interface, interfacial characteristics, interfacial bonding energy, fracture energy, and damage evoluation law under different loading temperatures and strain rates.

### 3.1. Interfacial Mechanical Response

#### 3.1.1. Temperature Effect

**Figure 4:**
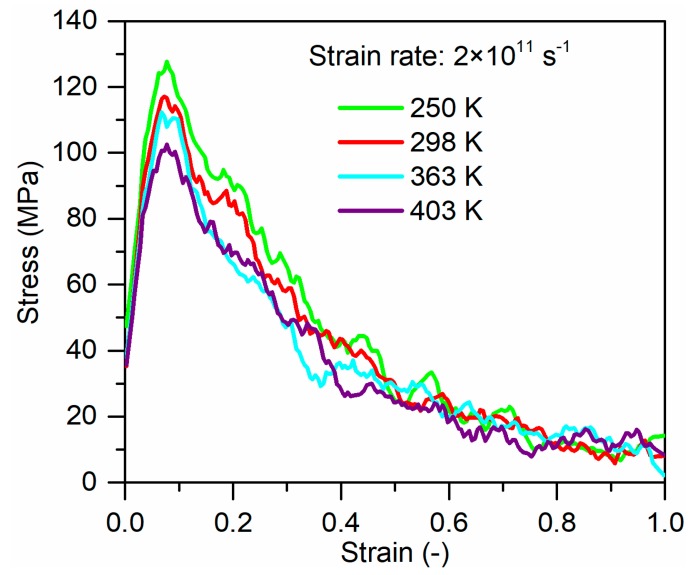
Tensile stress-strain curves of glass fiber/PP composites at different loading temperatures.

**Figure 5:**
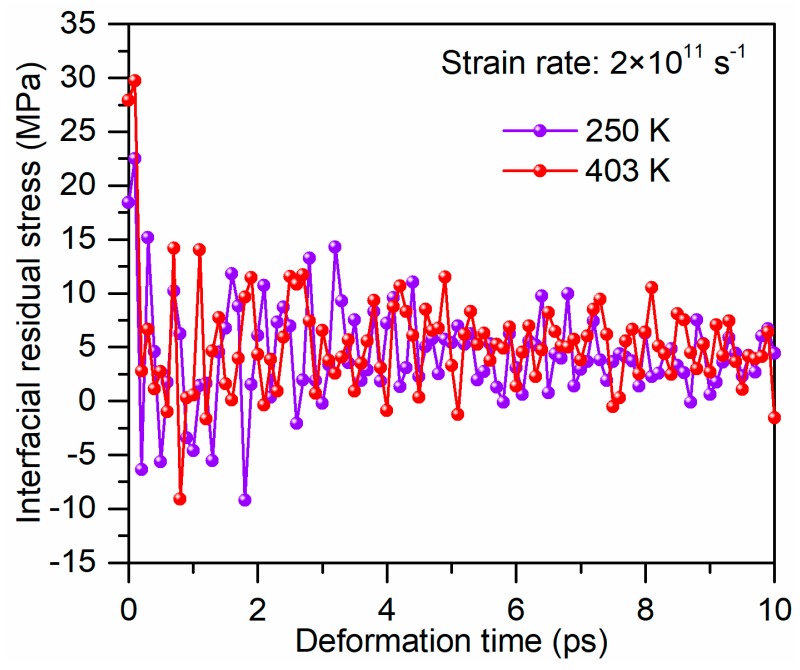
Interfacial residual stress variation during tensile deformation at different loading temperatures.

Figure 4 presents the stress–strain curves for the separation between glass fiber and PP under the loading temperatures ranging from 250 to 403 K. All curves comprise two basic regions, namely, a limited linear elastic region in an initial stage followed by yield and a softening region. The interfacial strength decreases slightly with increasing temperature. At 403 K the interfacial strength decreases to approx. 22% of that at 250 K. The previous studies reported that the reduction in the interfacial strength at the fiber–matrix interface is caused by the decrease in mechanical properties of matrix and the relaxation of static friction at the interface [16,32,33]. The static friction is influenced by the residual stress caused by the mismatch in thermal properties and Poisson’s ratio between fiber and matrix. To explore the influence of residual stress on the interfacial strength, the residual stress at the fiber–PP interface was calculated at different temperatures, as shown in Figure 5. It can be seen that loading temperature has a weak influence on the residual stress at the glass fiber–PP interface. Therefore, it can conclude that the decrease in the mechanical properties of PP is primarily responsible for the changes in interfacial strength at elevated temperatures.

**Figure 6:**
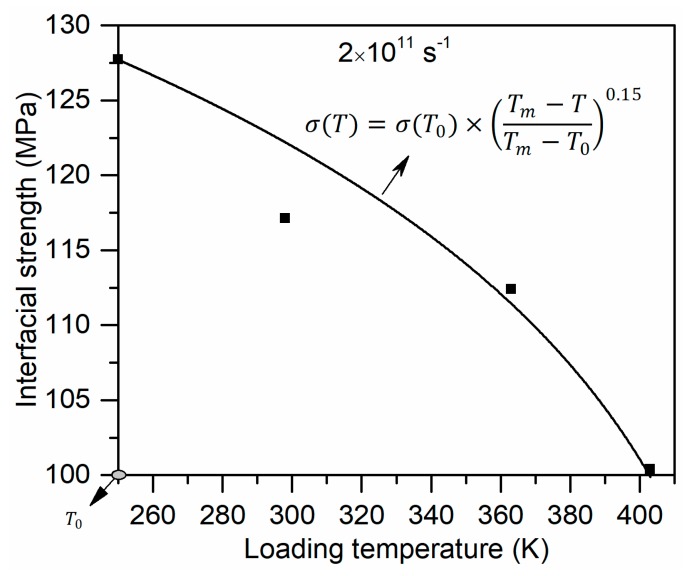
The interfacial strength as a function of loading temperature.

A function was proposed in our previous work [27] to assess the dependence of mechanical properties of bulk glass fiber/PP composites on loading temperature, which is in the range of from room temperature (reference temperature) to 90 °C. It was written as

(7) $$M\left(T\right)=M\left(T_{0}\right)\times {\left(\frac{T_{m}-T}{T_{m}-T_{0}}\right)}^{n}$$

where $M\left(T_{0}\right)$ is the mechanical properties at the reference temperature $T_{0}$; M(T) is the mechanical properties at elevated temperature T; $T_{m}$ is the melting temperature of matrix. As seen in Figure 6, Equation (7) can also give a good description on the decaying tendency of the interfacial strength of the glass fiber–PP interface at the atomic scale. The interfacial shear strength at the glass fiber–PP interface was measured by the single fiber fragmentation test in our previous work [27]. It can be found that the orders of the experimentally-obtained interfacial strength and numerically-obtained interfaical strength are in good agreement. However, qualitatively, the reduction in interfacial strength of the fiber–PP interface at elevated temperatures deviates most from that observed in experiments. To be specific, at 363K, the interfacial strength obtained experimentally decreases to approximately 71% of that at 298 K. The significant gap between the MD simulation and experimental results in interfacial strength is probably caused by different loading mode. This aspect will be further investigated in future studies.

#### 3.1.2. Strain Rate Effect

**Figure 7:**
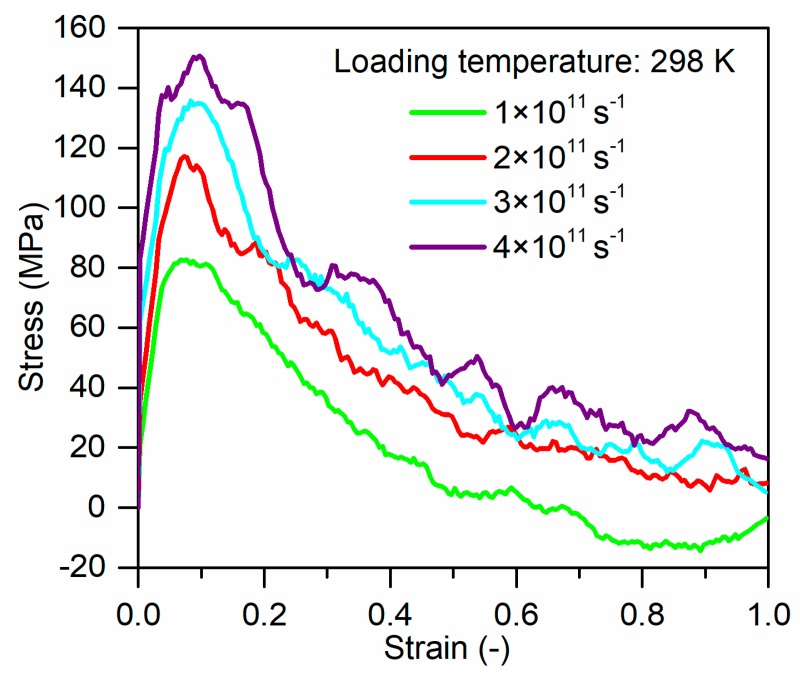
Tensile stress–strain curves of glass fiber/PP composites at different strain rates.

**Figure 8:**
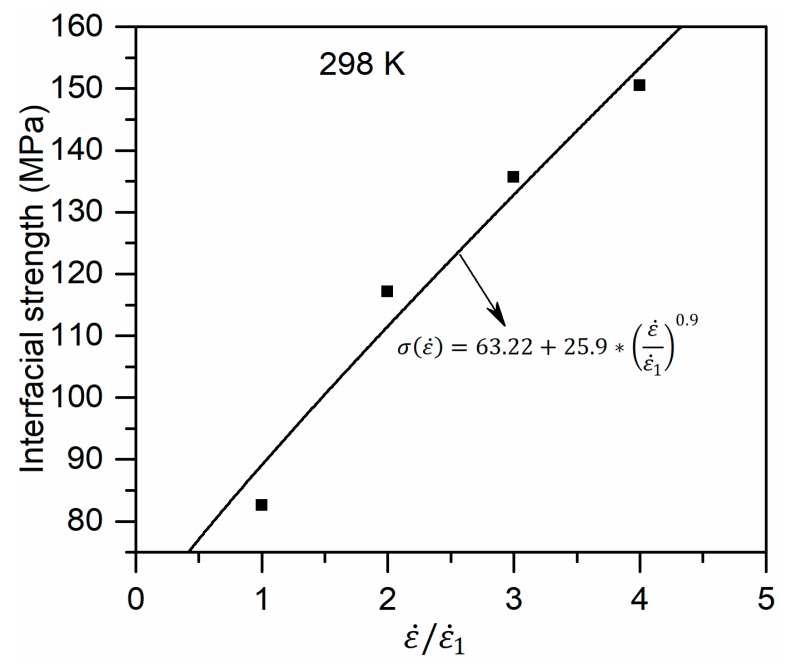
The interfacial strength as a function of strain rate.

Figure 7 shows the stress–strain curves for glass fiber/PP composites system at 298 K with the strain rates of 1 × 10^11^ s^−1^, 2 × 10^11^ s^−1^, 3 × 10^11^ s^−1^, and 4 × 10^11^ s^−1^. The stress–strain curves at different strain rates exhibit the same trend with the curves in Figure 4. The yield stress and elastic modulus increase with strain rate. The interfacial strengths obtained from the MD simulations are plotted in Figure 8. Interestingly, the interfacial strength can be well fitted with Equation (8), which is usually used to evaluate the strain rate effects on the macromechanical properties of glass fiber/PP composites. These trends are as expected and agree qualitatively with the experimental and computational theoretical results [12,34].

(8) $$M\left(\dot{\varepsilon }\right)=\alpha +\beta *{\dot{\left(\frac{\varepsilon }{\dot{\varepsilon_{1}}}\right)}}^{\gamma }$$

where M and $\dot{\varepsilon }$ are the overall mechanical properties and strain rate, respectively. α, β and γ are material constants, which can be determined through numerical fitting of MD data. ${\dot{\varepsilon }}_{1}$ indicates the strain rate at a reference state. In present study, the strain rate of 1 × 10^11^ s^−1^ was chosen as the reference state.

### 3.2. Interfacial Characteristics

**Figure 9:**
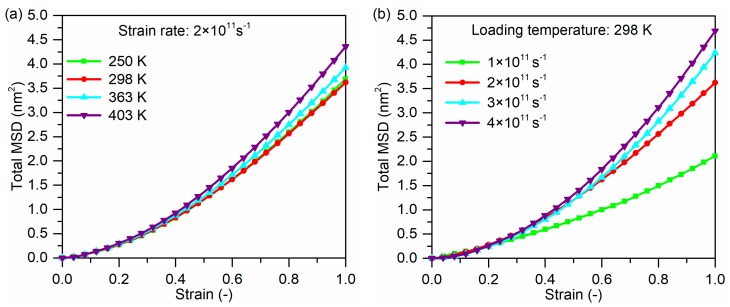
Mean square displacement (MSD) of PP molecules of glass fiber/PP composites models under: (**a**) different loading temperatures and (**b**) different strain rates.

**Figure 10:**
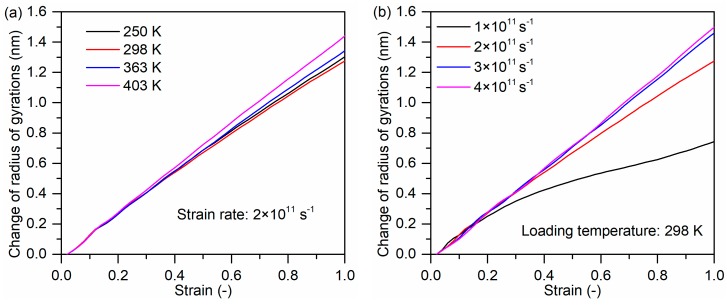
Change of the radius of gyration for PP molecules as a function of (**a**) loading temperatures and (**b**) strain rates.

The influence of loading temperature and strain rate on the MSD and change of $R_{g}$ for PP molecules were investigated, which are illustrated in Figure 9 and Figure 10, respectively. The total MSD increases slightly with increasing temperature because PP atoms posses enhanced thermal motion at elevated temperatures, as shown in Figure 9a. This trend corresponds well with a previous simulation study [35]. Meanwhile, the accelerated movements result in the increase of $R_{g}$, as demonstrated by the changes of $R_{g}$ in Figure 10a.

At the temperature of 298 K, the total MSD of PP molecules increases with increasing strain rate, as shown in Figure 9b. When the strain rate is higher, PP molecules do not have enough time to relax to fit the large deformation so that PP molecules possess higher MSD. This also can explain that the change of $R_{g}$ is more obvious at a higher strain rate in Figure 10b.

**Figure 11:**
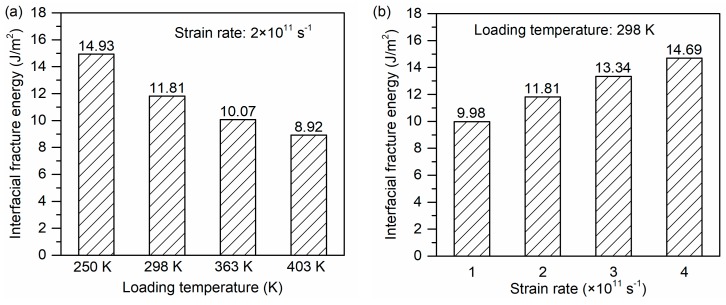
The interfacial fracture energy of glass fiber/PP composites under different loading conditions: (**a**) loading temperatures and (**b**) strain rates.

The interfacial fracture energy is assumed to be an indicator of the bonding strength between the glass fiber and PP. The influence of loading conditions on the interfacial fracture energy of glass fiber/PP composites is illustrated in Figure 11. As shown in Figure 11a, the loading temperature has a significant influence on the interfacial fracture energy of glass fiber/PP composites, which deceases with increasing loading temperature. The interfacial fracture energy at the 250 K case is the highest, followed by the 298 K case. At the loading temperature of 403 K, the fracture energy decreases to approximately 59.7% of that at 250 K. It indicates that the glass fiber–PP interface is deteriorated after the composites are exposed to elevated temperatures, which agrees well with the results reported by the previous studies [27,36].

Figure 11b gives the interfacial fracture energy of glass fiber/PP composites under different strain rates. It can be found that increasing strain rate contributes to a remarkable increase in the fracture energy, which shows the same tendency with the interfacial strength varying in strain rate. Specifically, the fracture energy at the strain rate of 4 × 10^11^ s^−1^ is increased by 147% compared to that at the strain rate of 1 × 10^11^ s^−1^. It can be understood that the atoms at high strain rate are less relaxed thus more energies are needed for the final fracture of composites.

### 3.3. Damage Mechanisms

**Figure 12:**
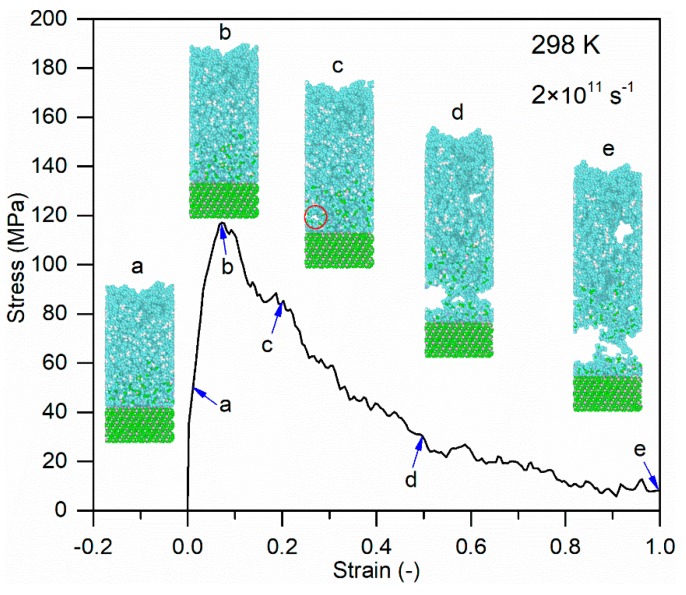
The configuration evolution of the glass fiber/PP material system during tensile loading at the loading temperature of 298 K and the strain rate of 2 × 10^11^ s^−1^.

Figure 12 indicates the configuration evolution of the glass fiber/PP material system at different tensile strains (1.0%, 7.1%, 20%, 50%, and 100%), in which the loading temperature of 298 K and the strain rate of 2 × 10^11^ s^−1^ case is employed for demonstration. The yield strain for this case is 7.1%. As shown in Figure 12, before the yield strain, the glass fiber–PP interface is relatively strong and the whole system stays stable. After the yield strain, the initial matrix crack can be found at the loading strain of 20%. With the further loading, the obvious cracks and rupture occur in the material system at the strain of 50%. Then, the crack become larger with the increase of strain until the interfacial fracture.

**Figure 13:**
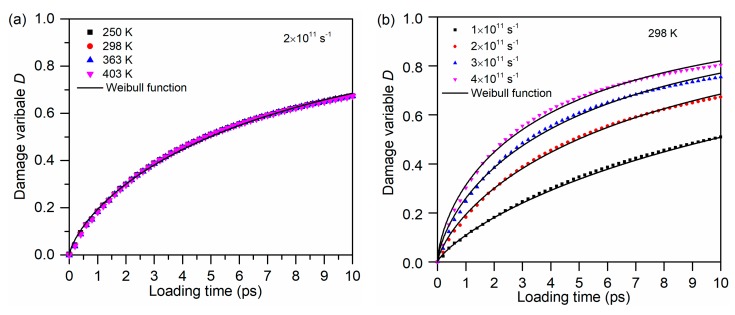
The damage variable of the glass fiber/PP material system during tensile loading under different loading conditions: (**a**) loading temperatures and (**b**) strain rates.

As illustrated in Section 2.6, the damage variable is employed to describe the damage evolution of glass fiber/PP material systems. The density profile of the glass fiber–PP interface at different loading temperatures and strain rates are given in supplementary material S1. According to Equation (6), the damage variables of the glass fiber/PP material system during tensile process were calculated, which are listed in Figure 13a,b. Moreover, the damage variable varying in loading time can be fitted by the Weibull function for all the loading cases. It indicates that the damage events in glass fiber/PP material systems at the micro level are of great randomness, which shows the same tendency with that in bulk composites at macro level. However, different from the strain rate, the loading temperature has no influence on the damage variable of the material system. It is probably because the accelerated thermal motion results in the higher local density in the interfacial region although the fiber–matrix interface is weak at elevated temperatures.

**Figure 14:**
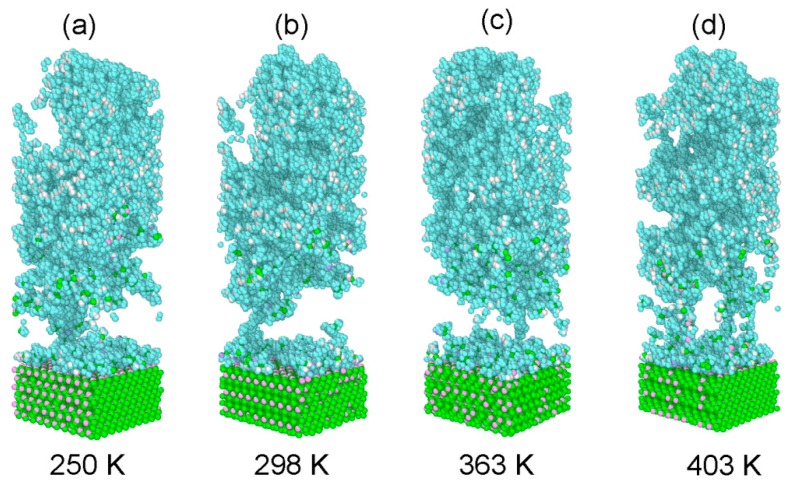
Snapshots of the deformation of the glass fiber/PP material system at the final strain of 100% at various loading temperatures with the strain rate of 2 × 10^11^ s^−1^.

Figure 14 gives snapshots of the deformation of the glass fiber/PP material system at the final strain of 100% under various loading temperatures. As can be seen, all the glass fiber/PP material system was damaged at the interfacial area regardless of the loading temperature. When the loading temperature is lower (250 K and 298 K), the adhesive failure and cohesive failure can be observed. However, at elevated temperatures, the cohesive failure is the primary fracture event. This behavior suggests that there is a competition between the failure mechanism and the weakening effect induced by elevated temperatures, which are same with the conclusions obtained from [13,36]. Elevated temperatures can bring about the reduction in matrix properties and interfacial strength at the glass fiber–PP interface. The cohesive failure occurred at elevated temperatures can be attributed to the weak strength of PP compared with that of the glass fiber–PP interface.

**Figure 15:**
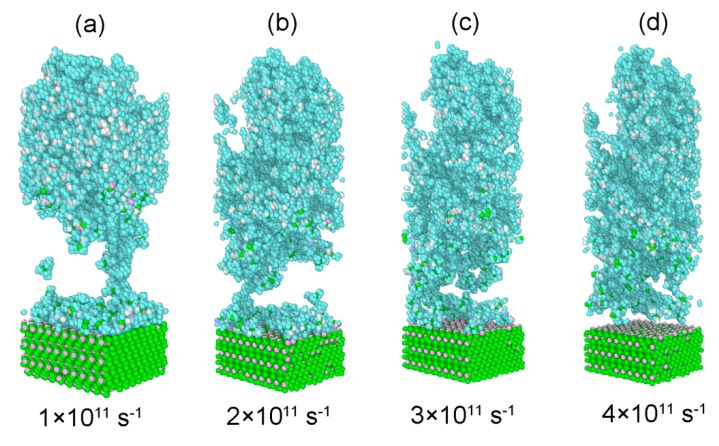
Snapshots of the deformation of the glass fiber/PP material system at the final strain of 100% at various strain rates with the loading temperature of 298 K.

The snapshots of the deformation of the glass fiber/PP material system at the final strain of 100% under various strain rates are shown in Figure 15. It was reported that the transverse tensile failure mode of unidirectional CFRTCs changes from matrix failure-dominant mode to interface failure-dominant mode with an increase of applied strain rate [37,38]. Our simulation results obtained the same conclusion. To be specific, at the strain rates of 1 × 10^11^ s^−1^ and 2 × 10^11^ s^−1^, the damage events are composed of PP rupture and glass fiber–PP interface debonding. While, with increasing strain rate, the primary failure mode is adhesive failure (Figure 15c,d). The reference reported by Pegoretti et al. has demonstrated that the shear strength at the glass fiber–matrix interface increases with strain rate [19], thus it can conclude that the strain rate dependency of PP is more pronounced than that of the glass fiber–PP interface. These findings are quite close to our earlier experimental results [12].

**Figure 16:**
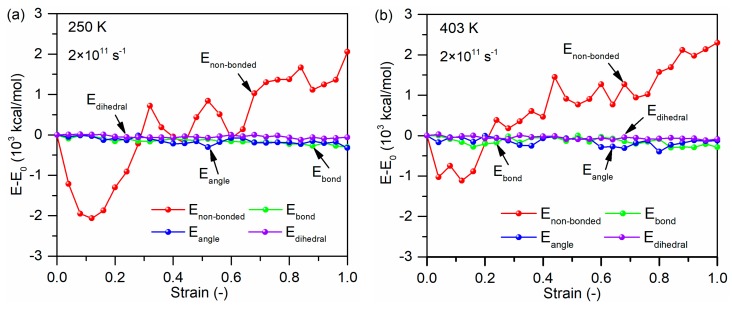
Potential energy decompositions of tensile process for the glass fiber/PP material system under the loading temperature of (**a**) 250 K and (**b**) 403 K.

Figure 16a,b displays the potential energy change versus tensile strain for the glass fiber–PP interface at the loading temperatures of 250 K and 403 K with the strain rate of 2 × 10^11^ s^−1^. As mentioned in Section 2.1.2, the potential energies are composed of bond stretching energy ($E_{\mathrm{bond}}$), angle bending energy ($E_{\mathrm{angle}}$), torsion potential energy ($E_{\mathrm{dihedral}}$), and non-bonded interaction energy ($E_{\mathrm{non}-\mathrm{bonded}}$). As shown, both the bond energy and angle energy exhibit an insignificant decrease with increasing tensile strain. The torsion energy experiences a negligible change during tensile process. It can also be observed that the non-bonded energy sharply increases after the yield strain due to breaking the non-bonded interactions between the chains. Furthermore, loading temperature has no obvious influence on the energy evolution curves of material system. The non-bonded interaction energy is responsible for the cohesive failure of the glass fiber/PP composites system under the tensile loading in the loading temperature ranges from 250 to 403 K.

**Figure 17:**
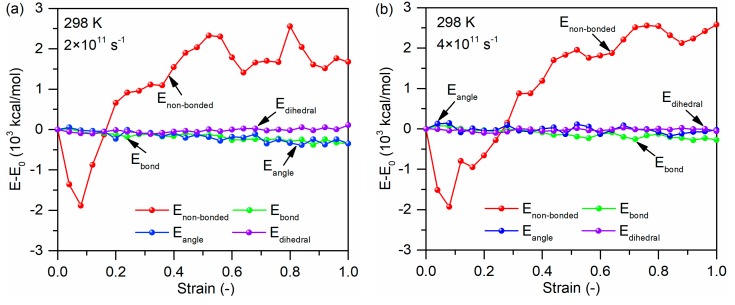
Potential energy decompositions of tensile process for the glass fiber/PP material system under the strain rate of (**a**) 2 × 10^11^ s^−1^ and (**b**) 4 × 10^11^ s^−1^.

Figure 17a,b shows the variation in internal energy as a function of strain rate for the glass fiber/PP material system at the loading temperature of 298 K. The non-bonded interaction energy plays a crucial role during the tensile deformation of the material system regardless of strain rate. With increasing strain rate, the changes in the non-bonded interaction energy is relative larger. This is because for the interface failure at high strain rate, both the non-bonded interactions between the oriented chains at the interface and interfacial non-bonded interactions need be failed in addition to non-bonded interactions between the chains in the matrix region.

## 4. Conclusions

In the present study, MD simulations were employed to study the interfacial mechanical behavior and failure mechanism of a glass fiber–PP interface under various loading temperatures and strain rates under uniaxial tensile loading. The obtained results were consistent with our previous experimental studies. The main conclusions of this work are as follows.

The interfacial strength and fracture energy at the glass fiber–PP interface decrease with increasing loading temperature, which is caused by the reduction in the mechanical properties of PP. However, qualitatively, the reduction in the interfacial strength of the fiber–PP interface at the elevated temperatures deviates most from that observed in experiments. It is probably due to the different loading modes adopted in experiment and MD simulations. This aspect will be further investigated in future studies.

At higher strain rate, the atoms are less relaxed, thus more fracture energies are needed for the final fracure, resulting in the higher interfacial strength at the glass fiber–PP interface.

It is demonstrated that the initial damage of the fiber–PP interface occurs randomly. The damage evolution with loading time for all the cases followes the Weibull distribution, which shows the same tendency with that in bulk composites at macro level.

The non-bonded interaction energy plays a crucial role during the tensile deformation of the glass fiber–PP interface. There is a competition between the failure mechanism and the weakening effect (enhanced effect) induced by elevated temperatures (strain rate). At elevated temperatures, the cohesive failure is a primary damage event due to the weak strength of PP compared with that of the glass fiber–PP interface. However, at a higher strain rate, the primary failure mode is interfacial failure because the strain rate dependency of PP is more pronounced than that of the glass fiber–PP interface.

## Figures and Tables

**Table 1:** Main parameters of polypropylene (PP) atomistic models.

Polymeric Material	Number of Chains	Degree of Polymerization	Total Amount of Atoms	Initial Density (g/cm^3^)	Box Size (nm)
PP	50	38	17176	0.9	5 × 5 × 5.9

## Supplementary Materials

The following are available online at https://www.mdpi.com/2073-4360/11/11/1766/s1, Figure S1: the density profile of glass fiber–PP interface at different loading temperatures; Figure S2: the density profile of glass fiber–PP interface at different strain rates.
