# Supplementary material for: The Effect of Temperature and Strain Rate on the Interfacial Behavior of Glass Fiber Reinforced Polypropylene Composites: A Molecular Dynamics Study
Source: Polymers (Basel). 2019 Oct 27;11(11):1766. doi: 10.3390/polym11111766 (PMC6918261; doi:10.3390/polym11111766)
Supplement: Supplementary file 1 [file polymers-11-01766-s001.pdf]

polymers

Supplementary Materials

The Effect of Temperature and Strain Rate on The Interfacial Behavior of Glass Fiber Reinforced Polypropylene Composites: A Molecular Dynamics Study

Muhan Zhang [1], Bingyan Jiang [1], Chao Chen [1], Dietmar Drummer [2] and Zhanyu Zhai [1,*]

[1] State Key Laboratory of High Performance and Complex Manufacturing, Light Alloy Research Institute, Central South University, Lushan South Road 932, 410083 Changsha, China; 183811004@csu.edu.cn (M.Z.); zzyu1021@hotmail.com (B.J.); profchenchao@163.com (C.C.)

[2] Friedrich-Alexander-University Erlangen-Nürnberg, Institute of Polymer Technology, Am Weichselgarten 9, D-91058 Erlangen, Germany; drummer@lkt.uni-erlangen.de (D.D.)

* Correspondence: zhanyuzhai@csu.edu.cn; Tel.: +xx-xxxx-xxx-xxxx

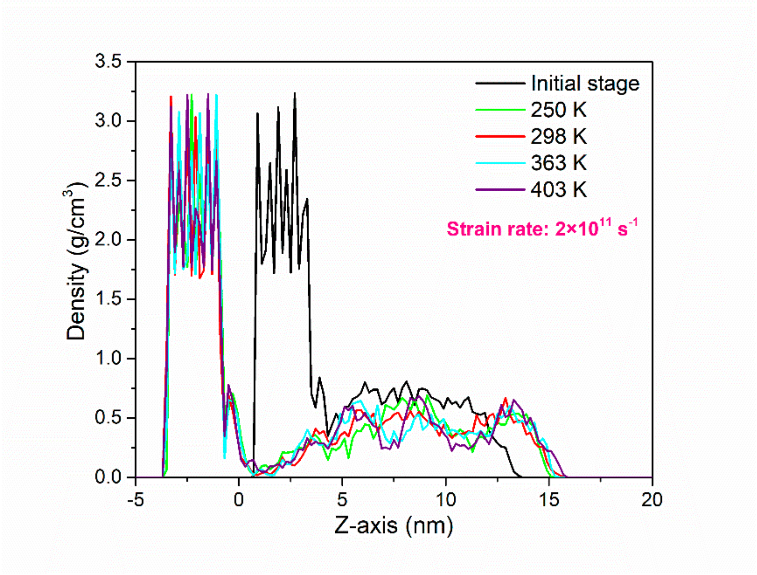

Figure S1. The density profile of glass fiber-PP interface at different loading temperatures.

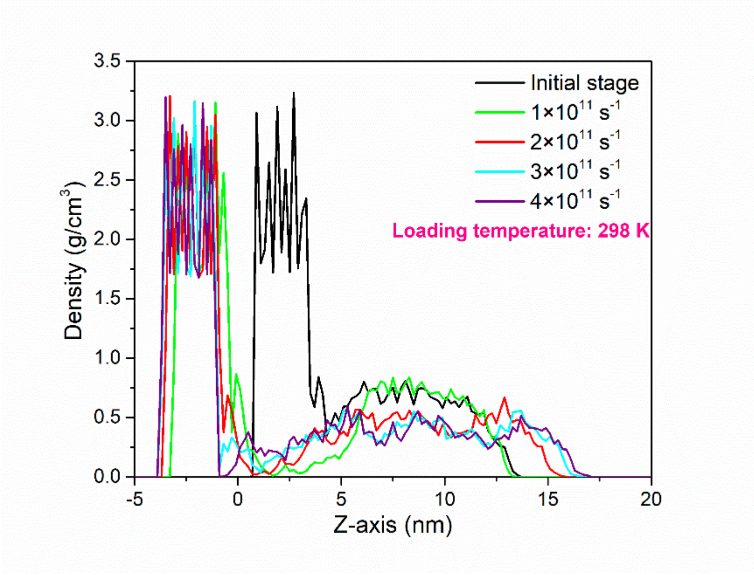

Figure S2. The density profile of glass fiber-PP interface at different strain rates.
